# Association of Cigarette Smoking With Pulmonary Embolism and Healthcare Utilization Following Hip and Knee Versus Shoulder Arthroscopy

**DOI:** 10.7759/cureus.110603

**Published:** 2026-06-10

**Authors:** Ronak J Mahatme, Shawn A Moore, Priyanka Parameswaran, Anish Gangavaram, Nishanth Muthusamy, Samuel K Gerak, David L Bernholt, Brian M Grawe

**Affiliations:** 1 Department of Orthopaedics, University of Cincinnati College of Medicine, Cincinnati, USA; 2 College of Medicine, University of Cincinnati, Cincinnati, USA; 3 Department of Orthopaedic Surgery, Cleveland Clinic Foundation, Cleveland, USA; 4 Department of Orthopaedic Surgery, University of Cincinnati College of Medicine, Cincinnati, USA

**Keywords:** cigarette smoking, hip arthroscopy, knee arthroscopy, pulmonary embolism (pe), shoulder arthroscopy

## Abstract

Background: Cigarette smoking is a well-established risk factor for adverse surgical outcomes due to its prothrombotic, inflammatory, and cardiopulmonary effects. However, its impact on short-term complications following arthroscopic surgery remains poorly defined. The purpose of the study is to evaluate the association between cigarette smoking and 90-day postoperative complications after shoulder, hip, and knee arthroscopic procedures.

Methods: Using a multicenter database, TriNetX, patients undergoing arthroscopic shoulder, hip, or knee procedures were retrospectively identified. Smokers were propensity score-matched 1:1 to nonsmoking controls by age, sex, comorbidities, and type of arthroscopic procedure. Ninety-day rates of venous thromboembolism (VTE), deep vein thrombosis (DVT), pulmonary embolism (PE), emergency department (ED) visits, hospital readmissions, postoperative infection, and wound dehiscence were compared between smokers and nonsmokers. Risk ratios (RRs) with 95% confidence intervals (CIs) were calculated.

Results: Among 18,948 shoulder arthroscopy patients, smoking was not associated with increased risk of VTE (RR, 1.086; 95% CI, 0.687-1.717; p = 0.815), DVT (RR, 1.037; 95% CI, 0.612-1.758; p = 1.000), or PE (RR, 1.000; 95% CI, 0.464-2.156; p = 1.000). Smokers demonstrated higher ED utilization (RR, 1.627; 95% CI, 1.435-1.844; p < 0.001), but no significant differences in readmission, infection, or wound complications. Among 24,196 hip/knee arthroscopy patients, smoking was not associated with overall VTE risk (RR, 1.103; 95% CI, 0.839-1.450; p = 0.527) or DVT (RR, 0.989; 95% CI, 0.737-1.326; p = 1.000), but was linked to an increased risk of PE (RR, 1.938; 95% CI, 1.060-3.540; p = 0.041). Cigarette smoking was further associated with higher ED visits (RR, 1.775; p < 0.001), readmissions (RR, 2.250; p < 0.001), and postoperative infections (RR, 1.525; p = 0.046) for knee and hip arthroscopy patients.

Conclusion: Cigarette smoking was not associated with increased overall VTE risk after shoulder arthroscopy but was linked to higher ED utilization. In lower extremity arthroscopy, smokers demonstrated an association with increased risk of PE, healthcare utilization, and infection. These findings highlight the need for targeted perioperative counseling and risk reduction strategies in patients who smoke undergoing arthroscopy.

## Introduction

Although cigarette smoking rates have declined in recent decades, cigarette use remains a major public health concern in the United States [1]. Smoking is most widely recognized for its association with lung cancer and other neoplasms, but it also contributes to a broad range of adverse health outcomes, including poor cardiovascular health, prothrombotic changes, and impaired inflammatory and wound-healing processes [2,3]. These systemic effects have led to a longstanding interest in the role of cigarette smoking within surgical populations.

The relationship between cigarette smoking and venous thromboembolism (VTE) has been studied in general medical and surgical patients, as well as in arthroplasty cohorts, where smoking has been associated with increased thromboembolic disease and wound complications [4,5]. However, the impact of smoking on outcomes after arthroscopic procedures remains unclear. Compared with arthroplasty, arthroscopy is associated with shorter operative times, lower physiologic stress, and routine outpatient pathways, contributing to a substantially lower baseline risk of VTE [6,7]. Despite these facts, the large volume of arthroscopic procedures performed annually means that even modest increases in complication rates among smokers may have important clinical implications [8,9]. Furthermore, it is unknown whether the effect of smoking on VTE rates differs by anatomic location, as lower extremity procedures may confer greater risk than upper extremity procedures due to postoperative mobility differences [10].

The purpose of the present study was to evaluate the association between cigarette smoking and postoperative complications following shoulder, hip, and knee arthroscopy in adults aged 18 and over. The primary outcome was overall VTE within 90 days postoperatively. Secondary outcomes included deep vein thrombosis (DVT), pulmonary embolism (PE), emergency department (ED) visits, readmission, postoperative infection, and wound complications. We hypothesized that smokers would demonstrate higher rates of postoperative VTE and related complications, particularly following lower extremity arthroscopy.

## Materials and methods

The data used in this retrospective cohort study were collected on August 6, 2025, from the TriNetX Research Network [11], which provided access to electronic medical records (diagnoses, procedures, medications, laboratory values, genomic information) from over 150 million patients from 102 healthcare organizations. TriNetX, LLC (Cambridge, MA, USA) [11] is compliant with the Health Insurance Portability and Accountability Act (HIPAA) and applicable data privacy regulations, and all data accessed through the platform is de-identified in accordance with federal standards. Because this study used only de-identified patient records and did not involve the collection, use, or transmittal of individually identifiable data, this study was exempted from Institutional Review Board approval.

Four cohorts of patients aged 18 years or older at the time of elective arthroscopic orthopaedic sports procedures were identified through a query of the TriNetX database using Current Procedural Terminology (CPT; a standardized procedural coding system maintained by the American Medical Association) and International Classification of Diseases, 10th Revision, Clinical Modification (ICD-10-CM; a standardized diagnostic coding system maintained by the Centers for Disease Control and Prevention) codes (see Appendices) [12,13]. The following four cohorts were identified: (1) shoulder arthroscopy with a history of cigarette smoking within one month prior to surgery, (2) shoulder arthroscopy without a history of cigarette smoking any time prior to and up to three months after surgery, (3) hip or knee arthroscopy with a history of cigarette smoking within one month prior to surgery, and (4) hip or knee arthroscopy without a history of cigarette smoking any time prior to and up to three months after surgery. Patients diagnosed with polytrauma within three months prior to surgery were excluded. Additionally, patients were excluded if they had a diagnosis of osteoporosis with a current pathological fracture, blood/coagulation disorders, long-term use of anticoagulants, neoplasms, acute or chronic kidney failure, prior VTE, or thrombophilia (see Appendices).

Propensity score matching was performed using a 1:1 greedy nearest-neighbor algorithm without replacement. A caliper of 0.1 pooled standard deviations was utilized to ensure close covariate balance. Propensity scores were estimated using a multivariable logistic regression, including age at time of surgery, sex, race, body mass index, diabetes, chronic obstructive pulmonary disease, and type of arthroscopic procedure. Standardized mean differences (SMDs) were used to assess covariate balance, with values <0.10 indicating appropriate balance. While propensity score matching reduces measured confounding, residual confounding may be present due to unmeasured variables. Only patients with complete data for all matching variables were included, and no imputation was performed.

Following matching, each shoulder arthroscopy cohort included 9,474 patients, and each hip/knee arthroscopy cohort included 12,098 patients. All covariates were appropriately balanced as indicated by SMDs <0.10 (Tables 1, 2).

**Table 1:** Patient Demographics and Propensity Score Matching of Cigarette and Control Patients Undergoing Shoulder Arthroscopy SMD: standardized mean difference

Characteristic	Unmatched Cohort			Matched Cohort		
	Shoulder Cigarette (% of Cohort)	Shoulder Control (% of Cohort)	SMD	Shoulder Cigarette (% of Cohort)	Shoulder Control (% of Cohort)	SMD
Total	9,475 (100%)	213,963 (100%)	-	9,474 (100%)	9,474 (100%)	-
Age at Index	50.2 ± 12.0 (100%)	50.9 ± 14.8 (100%)	0.059	50.2 ± 12.0 (100%)	50.2 ± 12.0 (100%)	0.005
Demographics						
White individuals	7,042 (74.3%)	155,520 (74.6%)	0.005	7,042 (74.3%)	7,072 (74.6%)	0.007
Black or African American individuals	1,022 (10.8%)	19,025 (9.1%)	0.056	1,022 (10.8%)	1,020 (10.8%)	0.001
Asian individuals	149 (1.6%)	5,259 (2.5%)	0.067	149 (1.6%)	145 (1.5%)	0.003
American Indian or Alaska Native individuals	60 (0.6%)	965 (0.5%)	0.023	60 (0.6%)	58 (0.6%)	0.003
Native Hawaiian or Other Pacific Islander individuals	82 (0.9%)	1,174 (0.6%)	0.036	82 (0.9%)	77 (0.8%)	0.006
Female	3,358 (35.4%)	78,220 (37.5%)	0.043	3,358 (35.4%)	3,357 (35.4%)	<0.001
Male	5,604 (59.1%)	121,918 (58.5%)	0.014	5,604 (59.2%)	5,605 (59.2%)	<0.001
Diagnosis						
Body mass index (BMI) 30-39, adult	1,466 (15.5%)	19,689 (9.4%)	0.183	1,465 (15.5%)	1,463 (15.4%)	0.001
BMI 40 or greater, adult	494 (5.2%)	6,568 (3.1%)	0.103	493 (5.2%)	503 (5.3%)	0.005
Diabetes mellitus	1,566 (16.5%)	23,750 (11.4%)	0.149	1,565 (16.5%)	1,589 (16.8%)	0.007
Other chronic obstructive pulmonary disease	1,253 (13.2%)	4,482 (2.1%)	0.425	1,252 (13.2%)	1,250 (13.2%)	0.001
Procedure						
Arthroscopy, shoulder, surgical	9,407 (99.3%)	207,312 (99.4%)	0.013	9,406 (99.3%)	9,421 (99.4%)	0.020
Arthroscopy, shoulder, diagnostic, with or without synovial biopsy (separate procedure)	104 (1.1%)	2,238 (1.1%)	0.002	104 (1.1%)	80 (0.8%)	0.026

**Table 2:** Patient Demographics and Propensity Score Matching of Cigarette and Control Patients Undergoing Hip and Knee Arthroscopy SMD: Standardized mean difference

Characteristic	Unmatched Cohort			Matched Cohort		
	Hip/Knee Cigarette (% of Cohort)	Hip/Knee Control (% of Cohort)	SMD	Hip/Knee Cigarette (% of Cohort)	Hip/Knee Control (% of Cohort)	SMD
Total	12,101 (100%)	353,487 (100%)	-	12,098 (100%)	12,098 (100%)	-
Age at Index	44.2 ± 12.8 (100%)	43.7 ± 15.7 (100%)	0.038	44.2 ± 12.8 (100%)	44.3 ± 13.0 (100%)	0.009
Demographics						
White individuals	8,668 (71.6%)	241,661 (72.0%)	0.008	8,666 (71.6%)	8,678 (71.7%)	0.002
Black or African American individuals	1,266 (10.5%)	28,860 (8.6%)	0.063	1,266 (10.5%)	1,287 (10.6%)	0.006
Asian individuals	196 (1.6%)	9,782 (2.9%)	0.087	196 (1.6%)	192 (1.6%)	0.003
American Indian or Alaska Native individuals	103 (0.9%)	1,719 (0.5%)	0.041	103 (0.9%)	87 (0.7%)	0.015
Native Hawaiian or Other Pacific Islander individuals	116 (1.0%)	2,096 (0.6%)	0.038	115 (1.0%)	116 (1.0%)	0.001
Female	5,117 (42.3%)	150,678 (44.9%)	0.053	5,115 (42.3%)	5,076 (42.0%)	0.007
Male	6,172 (51.0%)	171,684 (51.2%)	0.003	6,171 (51.0%)	6,210 (51.3%)	0.006
Diagnosis						
Body mass index (BMI) 30-39, adult	2,041 (16.9%)	27,587 (8.2%)	0.263	2,038 (16.8%)	2,057 (17.0%)	0.004
BMI 40 or greater, adult	882 (7.3%)	10,541 (3.1%)	0.187	879 (7.3%)	922 (7.6%)	0.014
Diabetes mellitus	1,222 (10.1%)	20,338 (6.1%)	0.149	1,220 (10.1%)	1,208 (10.0%)	0.003
Other chronic obstructive pulmonary disease	1,006 (8.3%)	3,911 (1.2%)	0.341	1,003 (8.3%)	995 (8.2%)	0.002
Procedure						
Arthroscopy, hip, surgical	645 (5.3%)	25,016 (7.5%)	0.087	645 (5.3%)	613 (5.1%)	0.012
Arthroscopy, knee, surgical (1005652)	55 (0.5%)	1,644 (0.5%)	0.005	55 (0.5%)	44 (0.4%)	0.014
Arthroscopy, knee, surgical (1005657)	10,651 (88.0%)	282,361 (84.1%)	0.112	10,648 (88.0%)	10,694 (88.4%)	0.012
Arthroscopy, hip, diagnostic with or without synovial biopsy (separate procedure)	22 (0.2%)	473 (0.1%)	0.010	22 (0.2%)	14 (0.1%)	0.017
Arthroscopy, knee, diagnostic, with or without synovial biopsy (separate procedure)	219 (1.8%)	5,266 (1.6%)	0.019	219 (1.8%)	185 (1.5%)	0.022
Arthroscopically aided anterior cruciate ligament repair/augmentation or reconstruction	2,431 (20.1%)	71,416 (21.3%)	0.029	2,431 (20.1%)	2,418 (20.0%)	0.003
Arthroscopically aided posterior cruciate ligament repair/augmentation or reconstruction	129 (1.1%)	1,687 (0.5%)	0.064	129 (1.1%)	112 (0.9%)	0.014

Outcomes were assessed within 90 days postoperatively and included VTE, DVT, PE, ED visits, hospital readmission, postoperative infections, and wound dehiscence (see Appendices).

Risk ratios (RRs) and 95% confidence intervals (CIs) were computed, and the complication rates were analyzed using the TriNetX system [11]. Categorical variables were assessed using the chi-squared test, while continuous variables were evaluated with independent t-tests. The level of statistical significance was set at P < 0.05. Because multiple postoperative outcomes were evaluated across two independent cohorts, findings were interpreted as exploratory, and no formal correction for multiple comparisons was applied.

## Results

Shoulder arthroscopy

Among 18,948 patients undergoing shoulder arthroscopic surgery, cigarette smoking was not associated with an increased risk of VTE (RR: 1.086; 95% CI: 0.687-1.717; p = 0.815), DVT (RR: 1.037; 95% CI: 0.612-1.758; p = 1.000), or PE (RR: 1.000; 95% CI: 0.464-2.156; p = 1.000).

**Figure 1:**
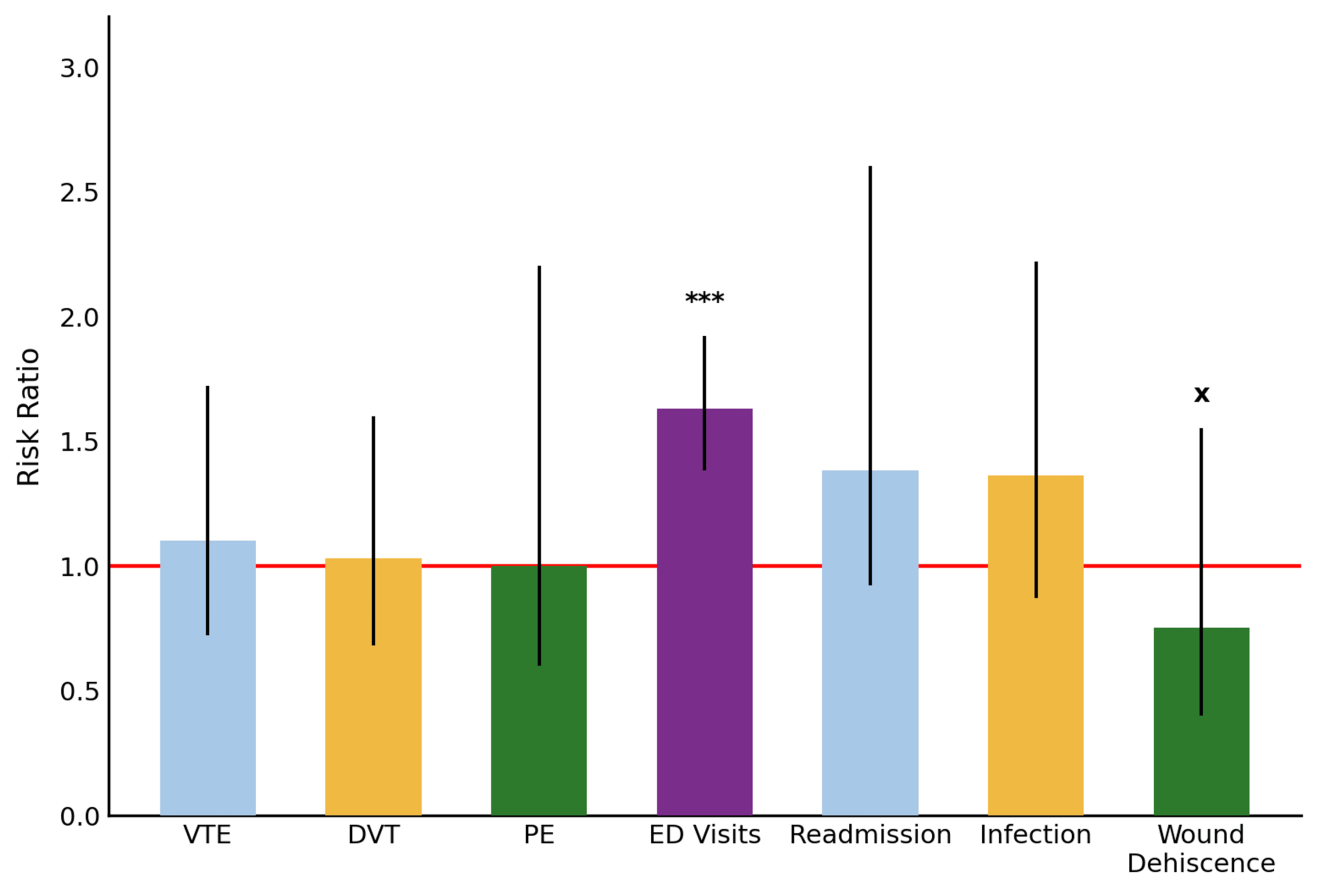
90-Day Outcomes Between Cigarette and Control Groups Following Shoulder Arthroscopy VTE: venous thromboembolism; DVT: deep vein thrombosis; PE: pulmonary embolism; ED: emergency department * < 0.05; ** < 0.01; ***< 0.001; x: ≤10 patients in at least one group due to TrinetX protection of patient information.

Patients who smoked demonstrated significantly higher rates of ED visits compared with nonsmoking controls (RR: 1.627; 95% CI: 1.435-1.844; p < 0.001). No significant associations were observed for hospital readmission (RR: 1.389; 95% CI: 0.758-2.544; p = 0.360), postoperative infection (RR: 1.367; 95% CI: 0.854-2.187; p = 0.234), or wound dehiscence (RR: 0.769; 95% CI: 0.337-1.753; p = 0.676) (Table 3 and Figure 1).

**Table 3:** 90-Day Outcomes Between Cigarette and Control Groups Following Shoulder Arthroscopy VTE: venous thromboembolism; DVT: deep vein thrombosis; PE: pulmonary embolism; ED: emergency department *Event counts are less than 11 and rounded due to TrinetX limitations in reporting small event count data to prevent patient deindividualization. ^p-values may not accurately represent significance as calculations are done with rounded patient populations.

Outcome	Cigarette Events (%)	Control Events (%)	Total	Risk Ratio	95% CI	P-value
VTE	38 (0.4)	35 (0.4)	9474	1.086	0.687-1.717	0.815
DVT	28 (0.3)	27 (0.3)	9474	1.037	0.612-1.758	1.000
PE	13 (0.1)	13 (0.1)	9474	1.000	0.464-2.156	1.000
ED Visits	610 (6.4)	375 (4.0)	9474	1.627	1.435-1.844	<0.001
Readmission	25 (0.3)	18 (0.2)	9474	1.389	0.758-2.544	0.360
Infection	41 (0.4)	30 (0.3)	9474	1.367	0.854-2.187	0.234
Wound Dehiscence	≤10* (0.1)	13 (0.1)	9474	0.769	0.337-1.753	0.676^

Hip or knee arthroscopy

Among 24,196 patients undergoing hip or knee arthroscopic surgery, cigarette smoking was not significantly associated with overall VTE risk (RR: 1.103; 95% CI: 0.839-1.450; p = 0.527) or DVT (RR: 0.989; 95% CI: 0.737-1.326; p = 1.000). However, smokers had a significantly higher risk of PE compared with nonsmokers (RR: 1.938; 95% CI: 1.060-3.540; p = 0.041).

**Figure 2:**
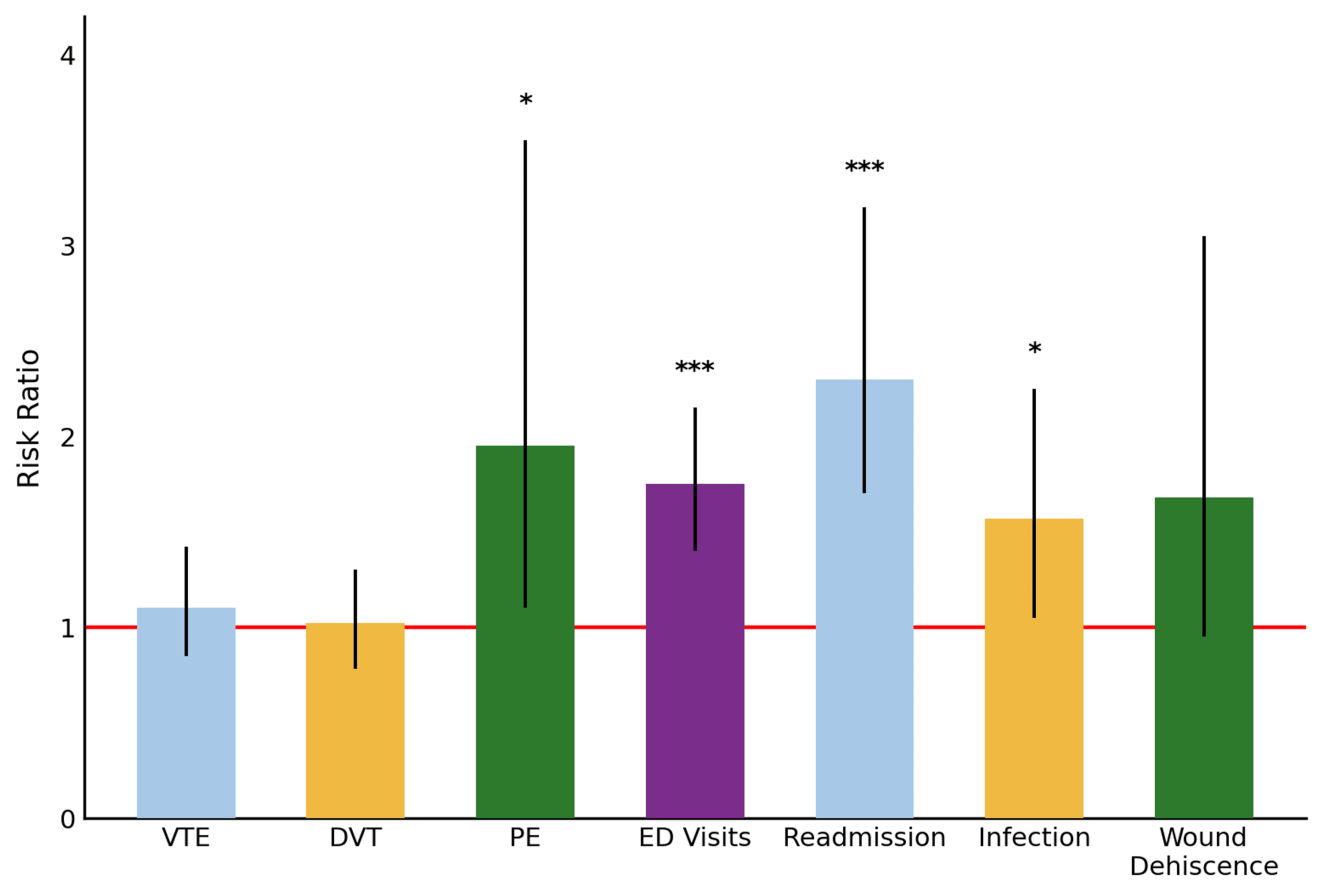
90-Day Outcomes Between Cigarette and Control Groups Following Hip/Knee Arthroscopy VTE: venous thromboembolism; DVT: deep vein thrombosis; PE: pulmonary embolism; ED: emergency department * < 0.05; ** < 0.01; ***< 0.001

Cigarette smoking was also associated with increased healthcare utilization and complications. Smokers demonstrated higher rates of ED visits (RR: 1.775; 95% CI: 1.598-1.973; p < 0.001), readmissions (RR: 2.250; 95% CI: 1.552-3.263; p < 0.001), and postoperative infections (RR: 1.525; 95% CI: 1.024-2.270; p = 0.046). There was no statistically significant difference in wound dehiscence (RR: 1.667; 95% CI: 0.930-2.988; p = 0.112) (Table 4 and Figure 2).

**Table 4:** 90-Day Outcomes Between Cigarette and Control Groups Following Hip/Knee Arthroscopy VTE: venous thromboembolism; DVT: deep vein thrombosis; PE: pulmonary embolism; ED: emergency department

Outcome	Cigarette Events (%)	Control Events (%)	Total	Risk Ratio	95% CI	P-value
VTE	107 (0.9)	97 (0.8)	12098	1.103	0.839-1.450	0.527
DVT	88 (0.7)	89 (0.7)	12098	0.989	0.737-1.326	1.000
PE	31 (0.3)	16 (0.1)	12098	1.938	1.060-3.540	0.041
ED Visits	909 (7.5)	512 (4.2)	12098	1.775	1.598-1.973	<0.001
Readmission	90 (0.7)	40 (0.3)	12098	2.250	1.552-3.263	<0.001
Infection	61 (0.5)	40 (0.3)	12098	1.525	1.024-2.270	0.046
Wound Dehiscence	30 (0.2)	18 (0.1)	12098	1.667	0.930-2.988	0.112

## Discussion

In this large propensity score-matched database study, cigarette smoking demonstrated varying associations with postoperative outcomes following arthroscopy. Contrary to our primary hypothesis, smoking was not associated with increased overall VTE rates in either shoulder or lower extremity arthroscopy cohorts. Furthermore, among patients undergoing shoulder arthroscopy, smoking was not associated with increased risk of DVT, PE, readmission, infection, or wound dehiscence. However, patients who smoked did have significantly greater rates of ED visits. In contrast, in the hip/knee arthroscopy cohort, smoking was associated with an increased incidence of PE in addition to higher rates of ED visits, readmission, and postoperative infection. There was no significant difference in rates of DVT or wound dehiscence based on smoking status for the hip/knee arthroscopy cohort. Given the low number of PE events and the lack of concordant DVT findings, thromboembolic findings should be interpreted with caution.

These findings suggest that the postoperative complication profiles associated with smoking may vary by surgical site, potentially reflecting differences in operative technique, patient positioning, post-operative restrictions or activity levels, or the inherently higher thrombotic risk of hip/knee procedures compared with shoulder procedures [14,15]. Smoking is a well-established prothrombotic factor through mechanisms including endothelial dysfunction, platelet activation, and impaired fibrinolysis [16,17]. Prior studies have shown smoking increases VTE risk following hip and knee arthroscopy, but not shoulder arthroscopy [15,18-21]. The low event rates of VTE following shoulder procedures may account for this difference, which aligns with our findings. Interestingly, smoking was associated with increased PE rates despite no differences in DVT or overall VTE. Given the expected biological relationship between these outcomes, this isolated association should be interpreted cautiously. Possible explanations include differential diagnostic coding, residual confounding, variation in event detection, or statistical variation [22]. Therefore, these findings should be considered exploratory and warrant further validation.

Beyond thromboembolic complications, smoking was associated with increased healthcare utilization. Shoulder arthroscopy patients who smoke had higher rates of ED visits, while hip/knee arthroscopy patients who smoke demonstrated increased ED visits and readmissions. These observations are consistent with prior studies showing that smokers are more likely to seek urgent care postoperatively, potentially due to higher baseline comorbidities, altered pain perception, or nicotine-related physiologic stress [23,24].

In hip/knee arthroscopy, the higher rates of readmission and infection among smokers may reflect longer operative times, more extensive soft tissue dissection, and the proinflammatory effects of smoking. Specifically, infections were significantly greater in the smoking cohort, consistent with prior literature demonstrating that smoking enhances infection risk following hip and knee procedures [25,26]. Smoking has been shown to impair neutrophil function, reduce tissue oxygenation, and compromise angiogenesis, thereby increasing infection susceptibility [27-29]. Interestingly, while prior studies suggest that smoking may increase infection risk following rotator cuff repair, our shoulder cohort did not demonstrate a significant association, likely due to the low baseline infection rates and limited surgical exposure inherent to arthroscopic shoulder procedures [30].

No significant differences in wound dehiscence were observed in either cohort. While smoking is a recognized risk factor for impaired wound healing, the small incisions used in arthroscopy likely mitigate this risk compared with open procedures.

This study has several limitations. As with all retrospective database analyses, misclassification of smoking status, procedures, and postoperative outcomes is possible. Smoking intensity, duration, cessation status, thromboprophylaxis protocols, postoperative weight-bearing restrictions, immobilization, operative duration, and procedure-specific characteristics were unavailable within the database and may contribute to residual confounding. Multiple outcomes were evaluated without formal correction for multiple comparisons, increasing the possibility of type I error. Additionally, the observed PE association was based on a relatively small number of events and occurred without corresponding differences in DVT or overall VTE rates, limiting mechanistic interpretation. While stratification by surgical site highlights key differences, there is also heterogeneity within each category, as the type of arthroscopic procedure performed can vary considerably across the knee, hip, and shoulder. We attempted to limit this variability by controlling the selection of CPT codes, focusing on overarching categories rather than including the full spectrum of arthroscopic codes. Finally, combining hip and knee arthroscopy introduces procedural heterogeneity as these operations differ in operative characteristics, postoperative mobility restrictions, rehabilitation protocols, and baseline thromboembolic risk. Despite these limitations, the large multicenter design and propensity score matching provide a broad assessment of smoking-associated postoperative outcomes following arthroscopy. While the findings do not support an association between smoking and overall VTE risk, the consistent increase in healthcare utilization observed across cohorts and selected complications in lower extremity arthroscopy support continued investigation into the perioperative effects of smoking.

## Conclusions

Cigarette smoking was not associated with increased overall VTE risk following shoulder or lower extremity arthroscopy. Among shoulder arthroscopy patients, smoking was associated with increased ED utilization but not higher rates of VTE or infection-related complications. In contrast, smokers undergoing hip or knee arthroscopy demonstrated increased risks of PE, postoperative infection, ED visits, and hospital readmission within 90 days of surgery. Given the observational design, low event counts, and absence of differences in overall VTE outcomes, thromboembolic findings should be interpreted cautiously and viewed as hypothesis-generating. Given the high volume of arthroscopic procedures performed annually, these findings support continued perioperative smoking counseling and motivate future procedure-specific investigation.

## Appendices

**Table 5:** CPT, ICD-9-CM, ICD-10-CM Codes for Query and Outcomes VTE: venous thromboembolism; DVT: deep vein thrombosis; PE: pulmonary embolism; CPT: Current Procedural Terminology; ICD-9-CM: International Classification of Diseases, Ninth Revision, Clinical Modification; ICD-10-CM: International Classification of Diseases, Tenth Revision, Clinical Modification

Diagnosis	Codes
Orthopaedic Arthroscopic Surgery	1005614, 29805, 1005648, 1005652, 1005657, 1005679, 29860, 29870, 29888, 29889
Osteoporosis With Current Pathological Fracture	M80
Blood Disorders & Anticoagulant Use	D55-D59, D60-D64, D65-D69, D70-D77, Z79.01, D68.5, D68.6
Kidney Failure	N17-N19
Neoplasms	C40-C41, D45, D46, D47, C81-C96
Polytrauma	T07
Nicotine Dependence, Cigarette Smoking	F17.21
Propensity Score Inputs	Age at Index, Female, Male, Race, Z68.3, Z68.4, E08-E13, J44, 1005614, 29805, 1005648, 1005652, 1005657, 1005679, 29860, 29870, 29888, 29889
Postprocedural Infection	T81.4
DVT	I82, T84.86, T84.81, I80.1, I80.2, 451.83
PE	I26
VTE	I26, I82, I80.1, I80.2, 451.83, T84.86, T84.81
Emergency Department Visits	1013712
Hospitalization	1013699
Wound Dehiscence	T81.30, T81.31, T81.32
